# 77 Case series of juvenile scleroderma from Nigeria

**DOI:** 10.1093/rheumatology/keac496.073

**Published:** 2022-10-07

**Authors:** A. D Faleye, I. N Akinwumi, I. J Akinola, B. H Olaosebikan, A. B Oladimeji, P. O Ubuane, O. O Adelowo

**Affiliations:** 1 Rheumatology Unit, Department of Internal Medicine, Lagos State University Teaching Hospital, Ikeja, Lagos, Nigeria; 2 Department of Paediatrics, Lagos State University Teaching Hospital, Ikeja, Lagos; 3 Department of Internal Medicine, Lagos State University College of Medicine, Ikeja, Lagos; 4 Department of Paediatrics, Lagos State University College of Medicine, Ikeja, Lagos

## Abstract

**Background:**

The word *scleroderma* means ‘hard skin’ that develops due to excessive accumulation of collagen. It is the third most frequent rheumatic disease in paediatric rheumatology after juvenile idiopathic arthritis and systemic lupus erythematosus. When it occurs in individual <16 years, it is called juvenile scleroderma. It is a rare disease that occurs in one per million children, documented to be more common in African adults with poorer survival states compared with Caucasians.

**Objectives:**

*To describe the clinical and laboratory characteristics of children with juvenile scleroderma seen in our clinic, thus, increasing its awareness*.

**Methods:**

Retrospective review of records of three children diagnosed with juvenile scleroderma at the paediatric Rheumatology Clinic of Lagos State University Teaching Hospital(LASUTH) between May 2018 to April 2022.

**Results:**

**Case 1**

A 12-year-old girl presented with skin tightness of the left hand and thigh of one year, contracture of the 3^rd^, 4^th^ and 5^th^ proximal interphalangeal joints, arthritis of the left wrist, skin induration of the dorsum of the left hand involving the 4^th^ and 5^th^ finger and extending to the wrist and skin and induration of the anterolateral aspect of the left thigh. Her blood tests showed an erythrocyte sedimentation rate (ESR) at 20 mm/h, an ANA titre of 1:2560, a negative anti-Scl 70/anti-centromere antibodies, a normal complete blood count and serum electrolytes/urea/creatinine. A diagnosis of linear scleroderma was made, the patient had prednisolone, methotrexate and folic acid, in addition to topical emollients. She improved clinically as observed during follow up visits six weeks after initiation of treatment but later defaulted from the clinic due to unknown reason.

**Case 2**

A 4-year-old girl presented with constitutional symptoms, swollen hands and feet, sclerodactyly, narrowing of oral aperture, ulcers at tips of the fingers, inflammatory pain of the large joints, hypopigmented macules on the face, trunk, abdomen and back and also abnormal capillaroscopy. Erythrocyte sedimentation rate was 22 mm/h with normal levels of electrolytes/urea/creatinine, thrombocytosis, ANA titre of 1:640 and negative anti-centromere and anti-U1RNP antibodies. A diagnosis of diffuse systemic sclerosis was made. She started prednisolone, methotrexate, nifedipine and omeprazole and was asked to do an ECG, an Echocardiogram, a spirometry and a chest HRCT but she couldn’t afford to do these investigations due to severe financial constraints. She was clinically stable for four months until she presented at the emergency room with sudden loss of consciousness and congestive cardiac failure. She died during resuscitation attempt.

**Case 3**

An 11-year-old girl known patient of haematology unit with sickle cell anaemia, presented with inflammatory arthritis of the small joints of the hands, elbows and knees of nine-month duration, sclerodactyly, contractures of the PIP of the fingers, narrowing of oral aperture, generalized hypopigmented macules and abnormal nailfold capillaroscopy. Investigation showed an ANA titre of 1:640, positive anti-Scl 70 antibodies, negative anti-centromere antibodies, ESR of 130 mm/h and thrombocytosis. The echocardiogram showed a normally structured heart with severe restriction on spirometry and features of interstitial lung disease on HRCT of the chest. She started mycophenolate mofetil, prednisolone and nifedipine. She later received 2 doses of rituximab due to slow clinical improvement. She is being followed up.

**Conclusion:**

Most data on scleroderma are from adult studies, we reported these three cases due to the rare occurrence of scleroderma in children, thus increasing its awareness.

